# Local Environment but Not Genetic Differentiation Influences Biparental Care in Ten Plover Populations

**DOI:** 10.1371/journal.pone.0060998

**Published:** 2013-04-17

**Authors:** Orsolya Vincze, Tamás Székely, Clemens Küpper, Monif AlRashidi, Juan A. Amat, Araceli Argüelles Ticó, Daniel Burgas, Terry Burke, John Cavitt, Jordi Figuerola, Mohammed Shobrak, Tomas Montalvo, András Kosztolányi

**Affiliations:** 1 Evolutionary Ecology Group, Hungarian Department of Biology and Ecology, Babeş-Bolyai University, Cluj Napoca, Romania; 2 MTA-DE “Lendület” Behavioural Ecology Research Group, Department of Evolutionary Zoology and Human Biology, University of Debrecen, Debrecen, Hungary; 3 Biodiversity Lab, Department of Biology and Biochemistry, University of Bath, Bath, United Kingdom; 4 Department of Animal and Plant Sciences, University of Sheffield, Sheffield, United Kingdom; 5 Department of Biology, College of Science, University of Hail, Hail, Kingdom of Saudi Arabia; 6 Department of Wetland Ecology, Estación Biológica de Doñana – CSIC, Sevilla, Spain; 7 Department of Biosciences, University of Helsinki, Helsinki, Finland; 8 Avian Ecology Laboratory, Department of Zoology, Weber State University, Ogden, Utah, United States of America; 9 Department of Biology, College of Science, Taif University, Taif, Saudi Arabia; 10 Servei de Vigilància i Control de Plagues Urbanes, Agència de Salut Pública de Barcelona, Barcelona, Spain; University of Lethbridge, Canada

## Abstract

Social behaviours are highly variable between species, populations and individuals. However, it is contentious whether behavioural variations are primarily moulded by the environment, caused by genetic differences, or a combination of both. Here we establish that biparental care, a complex social behaviour that involves rearing of young by both parents, differs between closely related populations, and then test two potential sources of variation in parental behaviour between populations: ambient environment and genetic differentiation. We use 2904 hours behavioural data from 10 geographically distinct Kentish (*Charadrius alexandrinus*) and snowy plover (*C. nivosus*) populations in America, Europe, the Middle East and North Africa to test these two sources of behavioural variation. We show that local ambient temperature has a significant influence on parental care: with extreme heat (above 40°C) total incubation (i.e. % of time the male or female incubated the nest) increased, and female share (% female share of incubation) decreased. By contrast, neither genetic differences between populations, nor geographic distances predicted total incubation or female's share of incubation. These results suggest that the local environment has a stronger influence on a social behaviour than genetic differentiation, at least between populations of closely related species.

## Introduction

In species with biparental care both the male and the female cooperate to raise the offspring, although the type and the extent of care provisioning often vary between sexes and taxa [1], [2]. Biparental care of young is an excellent model system to investigate how cooperation and conflict shape social behaviour [3], [4]. First, biparental care is a common behaviour that occurs in a wide range of taxa including insects, fishes, frogs, birds and mammals [5]–[9]. Second, biparental care consists of discrete recognizable components, such as incubation, brood attendance, shared protection and feeding of the young that can be easily quantified in both the field as well as in controlled laboratory conditions. Third, the outcome of the parental care, the number and quality of offspring, is a Darwinian measure of fitness, and thus directly tells us how successful the behaviour is [1], [10]. Finally, biparental care is one of the few aspects of life-histories and behavioural ecology that has been frequently investigated and manipulated in various ecological settings [2], [11], [12], and thus has the potential to reveal how diverse ecologies influence social behaviour.

Biparental care represents a careful balance between conflict and cooperation [3], [13]. We define cooperation as a demanding activity of the parents, which benefits both the acting individual and its partner, while aims to maximize their reproductive success [14]. On the one hand, by cooperating to rear young, parents tend to increase the survival chances of their offspring, especially in situations when one parent cannot fully compensate for the lack of the partner [1], [11]. For instance high predation risk, limited resource availability or intense competition may require both parents to successfully rear the young [9]. In addition sex-specific specialization of the adults in certain parental tasks can also promote the evolution of biparental care (e.g. in burying beetle *Nicrophorus vespilloides* the females feed the larvae, whereas males mainly clean the carcass from fungus and bacteria [15]). On the other hand, biparental care is prone to conflict [3], [16], [17], since the parents pay the costs of rearing (e.g. time, energy and mortality) individually, whereas both biological parents share the benefit of care (the young). A deserting parent that leaves its partner (and the young) may re-mate and gain enhanced reproductive success, whereas its mate may need to spend weeks on rearing the young to independence while experiencing reduced survival and/or missed breeding opportunities [18], [19]. Therefore, biparental care is reminiscent of public goods game, and each parent has the temptation to cheat [20].

Variation in parental behaviours may be due to environmental differences, genetic differences, or their interaction [12], [15], [21]. Biparental care is often observed in an extremely dry, cold or hot environment, where the optimal environment for the developing embryo is substantially different from the ambient environment and one parent cannot provide sufficient care on its own (harsh environment hypothesis, [8], [14], [22], [23]. Phylogenetic comparative analyses support the harsh environment hypothesis in certain taxa but refute it in others. For instance, small pools have limited resources for the developing tadpoles that facilitate biparental care in frogs [9]. In contrast Mank et al. [24] failed to identify any ecological correlates of biparental care in comparative analyses of bony fishes.

One of the fundamental patterns in evolution is that closely related populations and species resemble more to each other than to distant ones, which is usually attributed to their shared evolutionary history. This pattern applies to morphology, behaviour and life histories, as indicated by significant phylogenetic signals in these traits [25], [26], including parental care [27], although behavioural traits tend to exhibit lower phylogenetic signals than body size, morphological, life-history, or physiological traits. Since much of the variation thought to occur at deep phylogenetic levels [6], one may predict that closely related species exhibit more similar social traits than distantly related ones, independently from the environment.

Extant species are often segregated into multiple partially (or fully) isolated breeding populations. These populations are subject to genetic drift and/or divergent selection over time. The degree of isolation regarding genetic mixing is thus expected to explain some of the differences in social behaviour between species and populations [28], as evidenced by the significant genetic component in various behavioural traits [15], [21], [29]–[31]. Here we investigate a social behaviour, parental care, and quantify whether genetic and spatial distance between different populations may explain the behavioural differences observed among populations. By calculating spatial distances separating populations, we investigate whether the difference in parental behaviour is predicted by isolation-by-distance model. The isolation-by-distance model predicts higher phenotypic similarity between populations with spatial proximity, or higher level of genetic mixing [26].

Socio-phylogeography, whereby populations that exhibit different social behaviours are compared across a wide range of ecological environments, is a powerful approach to investigate the influences of both environmental variables and genetic composition on social behaviour [32]. By estimating the genetic distances between populations, it is also possible to test whether the extent of genetic differentiation correlates with differences in behaviour. Here we carry out such a study in small plovers *Charadrius* spp. Plovers are eminently suitable for phylogeographic analyses of parental behaviour for two major reasons. First, they have an unusually wide breeding distribution that ranges from the Arctic Circle in the north down to Tierra del Fuego, South Africa and New Zealand in the south [33]. The ecological conditions within this vast geographic range are very diverse; plovers breed in subarctic tundra, temperate zone grasslands, marine and inland coasts, as well as in high mountain habitats, deserts, semi-deserts and salt marshes. Second, plovers exhibit diverse mating and parental behaviours both within a single population as well as between populations: some are strictly monogamous and both the male and the female care for the offspring, whereas others are polygynous or polyandrous with uniparental (or a variable degree of biparental) care of the eggs and the young by the male or the female [34].

We investigate biparental care of eggs in two closely related plover species, the Kentish plover (*Charadrius alexandrinus*) and the snowy plover (*C. nivosus*). Until very recently, snowy plovers were included in the Kentish plover species, although recent molecular evidence suggests that the two species are paraphyletic [35]. Both species exhibit biparental care of the eggs since both the male and the female incubate the eggs and attend the nest: the males largely incubate at night whereas the females carry out most of daytime incubation [36], [37]. Using 10 geographically distinct breeding populations, we test whether biparental care of eggs varies between populations. Some of these populations exhibit different brood care patterns [34], although our focus in this paper is on incubation behaviour. We then investigate whether local environment, and/or genetic distance between populations predict parental behaviour.

Although parental behaviour has been investigated from various perspectives [12], [38], [39], and a suite of theoretical and empirical studies have revealed how life-history traits and ecology influence parental behaviour (reviewed by [1], [10], [40]), our work is important and novel for three reasons. First, we use a socio-phylogeographic approach and compare parental behaviour between geographically distant breeding populations. Our dataset covers a large geographic range (latitude: 15°N –53°N, longitude: 112°W –54°E), and therefore, allows us to test the responses of parents to an unusually wide range of environmental conditions. Second, although latitudinal variation in incubation behaviour has been investigated previously (e.g., [41]), our focus is on the behaviour of both parents, whereas previous works mainly focussed on a single sex. Our work is therefore relevant to a core evolutionary issue: conflict and cooperation in an ecological context. Third, we test the effects of both environment and genetic disparity on biparental care. Although the effects of nature and nurture are highly controversial on social behaviour [42], we are not aware of any comparable study that investigated both issues in wild populations using parental care as a model paradigm.

## Materials and Methods

### Parental behaviour

**Figure 1:**
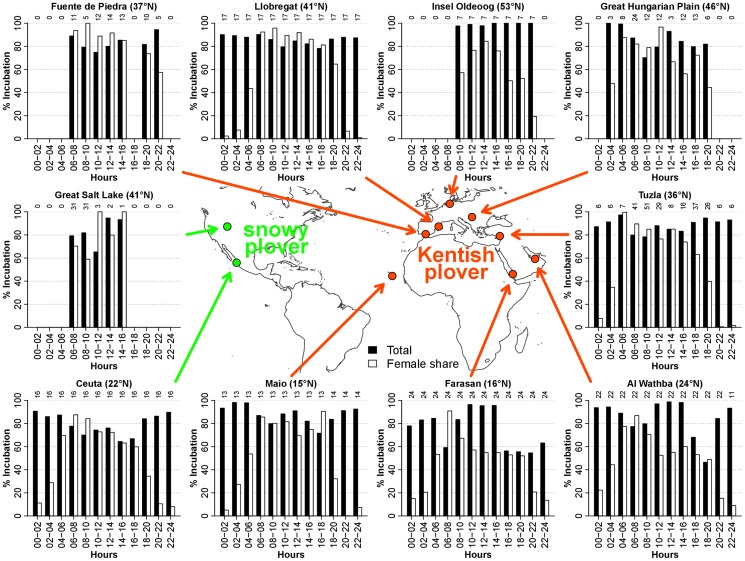
Total incubation by male and female (mean %, solid bars), and female share of incubation (mean %, open bars) in 10 plover populations over 12 time periods of the day. The number of nests for each time period is provided above the bars.

Incubation behaviour was recorded in 10 populations using transponder tags (4 populations), nest cameras (3 populations) or by direct observations (5 populations) (Table 1; in two populations two methods were used). The detailed methodologies are given by specific studies (see references in Table 1). In short, behavioural observations were carried out during daylight hours using a hide from sufficient distance to avoid disturbing the breeding bird. Nest cameras and transponder system were installed at nests, and they recorded behaviour for 24 hour periods. The error rate of automatic devices was low, approx 0.2% [37]. We define incubation as keeping the temperature of eggs within an optimal thermal interval for embryonic development, which involves both contact incubation (i.e. when the brood patch touches the eggs) and egg shading, which is exhibited in hot environments [14], [23], [43]. Data were available for 2904 hours of continuous records of incubation behaviour at 285 nests (Table S1, Fig. 1). We used two variables to quantify incubation behaviour: percent total incubation (% of time the eggs are incubated by the male or the female), and percent female share of incubation (% female share of % total incubation) in each specified time period (see bellow).

**Table 1:** Species, locality, country, breeding site (island (I) or mainland (M)), geographic coordinates and method of behaviour recording of 10 breeding plover populations for which we collected incubation data.

Population	Species	Locality	Country	Breeding site	Coordinates	Data collection method	Reference
1	Kentish plover	Oldeoog Island	Germany	I	53°45′N, 8°0′E	Observer	[44]
2	Kentish plover	Great Hungarian Plain	Hungary	M	46°,40′N, 19°10′E	Observer	T. Székely, unpublished data
3	Kentish plover	Delta del Llobregat	Spain	M	41°18′N, 2°8′E	Transponder	J. Figuerola, D. Burgas, T. Montalvo, unpublished data
4	Kentish plover	Fuente de Piedra	Spain	M	37°06′N, 04°45′W	Observer	[23]
5	Kentish plover	Tuzla	Turkey	M	36°42′N, 35°03′E	Observer and transponder	[37]
6	Kentish plover	Al Wathba	United Arab Emirates	M	24°16′N, 54°36′E	Camera	[43]
7	Kentish plover	Farasan Island	Saudi Arabia	I	16°48′N, 41°53′E	Transponder and camera	[14]
8	Kentish plover	Maio	Cape Verde	I	15°09′N, 23°13′W	Camera	T. Székely, A. Argüelles Tico, unpublished data
9	Snowy Plover	Great Salt Lake	USA	M	41°03′N, 112°06′W	Observer	J. Cavitt, unpublished data
10	Snowy Plover	Ceuta	Mexico	M	23°52′N, 106°55′W	Transponder	C. Küpper, unpublished data

To investigate the temporal pattern of incubation behaviour we divided the day into twelve 2-hour time periods following previous analyses of incubation [43], [14], and calculated % total incubation and % female share for each time period separately. Only observations that lasted for at least 30 minutes in a given 2-hour time period were included in the dataset. If for a given nest several records were available in the same time period from different days, we took their average and calculated the corresponding explanatory variable values, i.e. average ambient temperature, average clutch age (see below).

**Figure 2:**
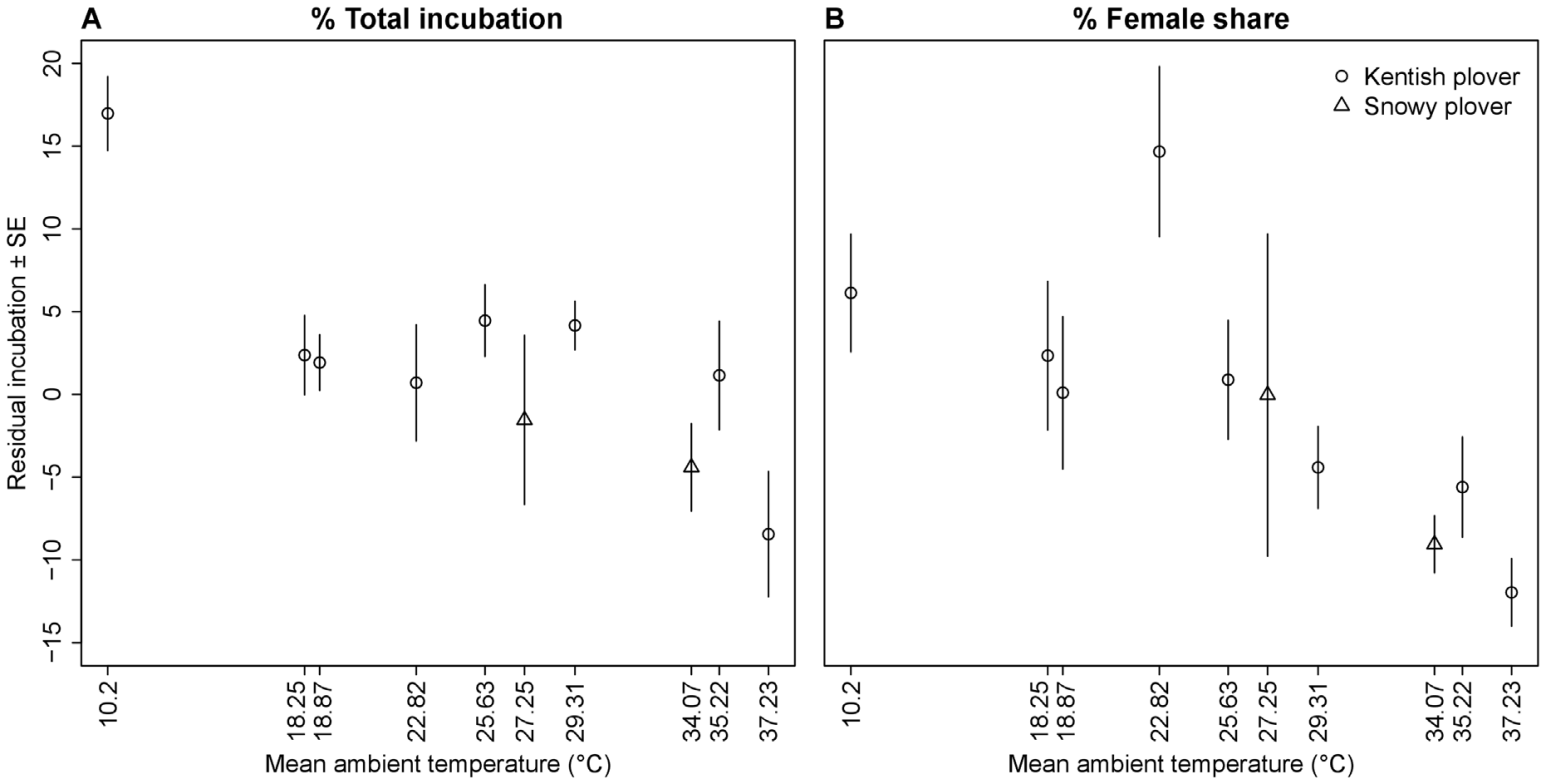
Residual total incubation and female share of incubation (mean ± SE) in relation to mean ambient temperature in 10 plover populations. Spearman rank correlations, total incubation: r_s_  = −0.661, p = 0.0440, female share: r_s_  = −0.891, p = 0.0014. After removing 2 population with extreme temperatures (Oldeoog and Farasan), the direction of both relationship remain although total incubation is no longer significant (total incubation: r  = −0.33, p = 0.4279, female share: r  = −0.86, p = 0.0107).

To illustrate population differences in total incubation and female share (Fig. 2A, B), we calculated mean residual total and female share of incubation, respectively, for each population. First, for each time period we calculated the difference between a given population's incubation (% total incubation, or % female share) and the mean incubation of all populations' data available for that time period. We then calculated the average of the residual differences for each population across all time periods as the mean residual incubation.

### Environmental and life-history data

We obtained data on ambient temperature separately for the 2-hour periods at all locations except for Oldeoog Island (Germany). In the latter population all observations were conducted on a single day, and only mean temperature was available for this day ([44], Table S1). Ambient temperature was measured at ground level except in Delta del Llobregat (Spain), where above ground temperature was recorded. Life history and behaviour of island-dwelling populations may be different from that of mainland populations [45], therefore we also investigated the effect of breeding site (island versus mainland) on incubation behaviour.

To test the effect of life history on incubation behaviour we used egg laying date and clutch age. Egg laying date was defined as the date of clutch completion which was either known for nests found during egg-laying, or was estimated in the field following Székely et al. [46]. Egg laying dates were standardized for each population separately to have a mean of zero and a standard deviation of one (z-transformation). Clutch age was calculated as the number of days elapsed between egg laying date and the date of behavioural observation. Since the parents' behaviour may be different in early incubation and/or near hatching of the eggs, we only included incubation records with clutch ages of minimum 3 and maximum 20 days. Kentish plover eggs hatch after 24–26 days of incubation [47].

### Genetic differentiation

Kentish and snowy plover populations exhibit gene flow across large geographic scales up to 10 000 km [48], [49]. The two species are phenotypically difficult to distinguish, and they were long considered to be the same species until significant genetic differences were demonstrated using microsatellites [35]. Because of the close relatedness between the populations in our study, microsatellites are highly suitable markers to estimate reproductive isolation. To quantify genetic differentiation between locations, we obtained blood samples of 25 presumably unrelated individuals from seven populations: Al Wathba, Ceuta, Farasan Island, Fuente de Piedra, Maio, Great Salt Lake and Tuzla. We genotyped all samples using 21 polymorphic autosomal microsatellites using the protocol in Küpper et al. [35]. Twelve microsatellite markers had known genome locations on available avian genome maps and were all found in non-coding areas [50], and therefore we assume that these markers are largely neutral. As a measure of genetic differentiation we calculated pairwise F_ST_ (fixation index) values between population pairs using the program ARLEQUIN version 3.1 [51]. We have also calculated genetic differentiation using a 427 bp mtDNA sequence [49]. Using mtDNA to estimate genetic differentiation provided fully consistent results with the microsatellite analyses (results not shown).

### Statistical analyses

Incubation behaviour may be consistent for a given nest, population or species, therefore we used a mixed model approach that included nests, populations and species as random factors. Both % total incubation and % female share were arcsine transformed, and used in mixed models with Gaussian error distribution. Time period was included as a fixed factor with 12 levels where each level represents a 2-hour time period. Environmental variables (ambient temperature, breeding site: island or mainland) and life-history variables (egg laying date, clutch age) were tested in two model groups to minimize data loss due to missing observations. In the first group of models (environmental variables) the fixed explanatory variables included time period, ambient temperature, breeding site as fixed variables, and time period × temperature interaction. The second group of models (life-history variables) included time period, egg laying date and clutch age. Time period × temperature was the only significant second order interaction (based on likelihood ratio statistics), and thus all other interactions were excluded from the models and not shown in the results.

Previous studies showed that ambient temperature has a quadratic effect on incubation behaviour [14], [43], therefore ambient temperature was included in the models as second degree orthogonal polynomial. To be consistent with analyses of total incubation, we kept the quadratic term in the % female share models although the quadratic term was not statistically significant in the latter models.

To test whether the effect of temperature on incubation behaviour varies between populations, the effect of temperature was estimated separately for each population using a random intercept and slope model. Unlike random intercept models, random intercept and slope models allow not only the intercept, but also the predicted slope to vary across the levels of the random factor. Since temperature data were not available for every time period in one population, the effect of environmental variables was tested in nine populations. To evaluate the significance of each predictor variable we used pairwise likelihood ratio based model comparisons.

We also investigated the effects of life-history and environmental variables for daytime and night time incubation separately following the aforementioned modelling approach. Daytime included time periods between 6.00 h – 18.00 h local standard (i.e. local time corrected for daylight savings), whereas night time included 18.00 h – 6.00 h.

To test the effect of genetic differentiation on incubation behaviour we used three approaches. First, we added species as random factor to models of environmental or life-history variables, and tested its effect using likelihood ratio statistics. Second, we calculated the pairwise mean incubation differences between pairs of populations for each time period, and took their average. Since the sign of difference (population A minus population B, or vice versa) is arbitrary, we took the absolute differences to calculate the mean % total incubation and % female share. We used two-sided Mantel tests to analyse the relationship between genetic differentiation (measured as pairwise F_ST_) and average behavioural differences in % total incubation and % female share between pairs of populations. F_ST_-values were calculated from presumably neutral genetic markers (see above), therefore with this method we target stochastic processes promoting genetic differentiation. Third, we computed geographic distances separating populations to test the isolation-by-distance model, and investigated their correlation with average behavioural differences in % total incubation and % female share between pairs of populations, using Mantel test. Since incubation behaviour was influenced by ambient temperature (see Results), we also calculated temperature corrected residual incubation behaviour, and tested the association between genetic differentiation and temperature-corrected incubation. The geographic distance matrix in km was computed using Geographic Distance Matrix Generator, version 1.2.3 [52]. Statistical analyses were carried out using R 2.14.0 [53].

### Ethical statement

The research lead to this publication has been carried out in full compliance to the ethical codes and legislation in each country in which it was performed. Blood sampling methods are given in Székely et al. (2008). Fieldwork and blood sampling was authorized by relevant authorities: Hungary ( Environmental Ministry and Kiskunság National Park), Spain (Catalan Ornithological Institute, Departament de Medi Ambient, Generalitat de Catalunya, The Consortium for the Protection and Management of the Natural Areas of Delta del Llobregat, Consejería De Medio Ambiente, Junta De Andalucía), Turkey (Turkish Ministry of National Parks, Tuzla Municipality and Governor of Karatas, Mr. E. Karakaya), United Arab Emirates (Environmental Agency), Saudi Arabia (Saudi Wildlife Authority), Republic of Cape Verde (Directorate Geral Ambiente), USA (US Fish and Wildlife Service, Bear River Migratory Bird Refuge, Utah Nature Conservancy, Weber State University, Animal Care and Use Committee), Mexico (Semarnat to Mr. Xico Vega, Pronatura Noroeste). Sampling in the latter population was carried out in collaboration with Dr. Blanca Estela Hernández Baños, Departmento de Biologia Evolutiva, Universidad Nacional Autonoma de Mexico, under permission of Semarnat.

## Results

### Incubation behaviour in different populations

Incubation behaviour (both % total incubation and % female share) was significantly different between plover populations, as indicated by the significant effect of the random intercept, and the random intercept and slope terms in mixed models of the full day (Table 2, 3, Fig. 1). Incubation behaviour remained significantly different between populations in models that also included time period, ambient temperature and breeding site (Table 2), or clutch age and egg laying date (Table 3). Population differences were persistent throughout the day, as these were significant for daytime as well as for night time % total incubation and % female share (Table 2, 3).

**Table 2:** The effects of environmental variables on total incubation (%) and female share of incubation (%).

	Full day		Daytime		Night time	
	(n_nests_ = 285; n_records_ = 1615)		(n_nests_ = 280; n_records_ = 968)		(n_nests_ = 150; n_records_ = 647)	
Model	?^2^ (df)	p	?^2^ (df)	p	?^2^ (df)	p
**Total incubation**						
Population (random intercept and slope)	69.77 (6)	<0.0001	59.44 (6)	<0.0001	9.72 (6)	0.1371
Population (random intercept)	14.88 (1)	0.0001	15.05 (1)	0.0001	6.67 (1)	0.0098
Time period	291.39 (33)	<0.0001	122.45 (15)	<0.0001	12.24 (15)	0.6610
Temperature	375.37 (29)	<0.0001	188.36 (17)	<0.0001	84.79 (17)	<0.0001
Slope difference between populations for temperature	54.89 (5)	<0.0001	44.39 (5)	<0.0001	3.05 (5)	0.6923
Period × temperature	194.17 (22)	<0.0001	78.79 (10)	<0.0001	4.52 (10)	0.9208
Temperature quadratic effect	91.49 (15)	<0.0001	22.29 (9)	0.0080	14.58 (9)	0.1031
Breeding site	0.32 (1)	0.5688	0.22 (1)	0.6395	0.16 (1)	0.6906
**Female share**						
Population (random intercept and slope)	23.5 (6)	0.0006	15.84 (6)	0.0147	11.51 (6)	0.0739
Population (random intercept)	6.68 (1)	0.0098	4.03 (1)	0.0446	0.00 (1)	1.0000
Time period	724.30 (33)	<0.0001	41.09 (15)	0.0003	227.01 (15)	<0.0001
Temperature	143.41 (29)	<0.0001	51.45 (17)	<0.0001	33.01 (17)	0.0113
Slope difference between populations for temperature	16.82 (5)	0.0049	11.81 (5)	0.0376	11.51 (5)	0.0422
Period × temperature	55.72 (22)	<0.0001	9.89 (10)	0.4499	15.60 (10)	0.1118
Temperature quadratic effect	18.11 (15)	0.2570	9.84 (9)	0.3638	17.32 (9)	0.0440
Breeding site	1.37 (1)	0.2424	1.34 (1)	0.2463	8.43 (1)	0.0037

Analysis for the full day (0–24 h), daytime (6–18 h) and night (18–6 h) data are shown separately.

Notes.

The full models included time period, ambient temperature, breeding site (mainland, island) and time period × temperature as fixed terms. The effect of temperature was estimated separately for each population by a random slope term. Nest ID was in the models as a random intercept term to control for pseudoreplication. Temperature was a second degree orthogonal polynomial. The significance of each predictor was assessed by eliminating it from the full model and comparing the fit of the two models using likelihood ratio test. Population effect was tested in two ways: (i) by removing the random intercept and slope term from the model, (ii) by replacing the random intercept and slope term with a random intercept term in the full model and removing this term. Temperature was tested by removing temperature, period × temperature and the random slope term for temperature from the model. The slope difference for temperature between populations was tested by removing the random slope term and keeping only the random intercept term in the model. The quadratic effect of temperature was tested by replacing the second degree orthogonal polynomial term with a linear term.

**Table 3:** The effects of life history variables on % total incubation and % female share.

	Full day		Daytime		Night time	
	(n_nests_ = 285; n_records_ = 1615)		(n_nests_ = 280; n_records_ = 968)		(n_nests_ = 150; n_records_ = 647)	
Model	?^2^ (df)	p	?^2^ (df)	p	?^2^ (df)	p
**Total incubation**						
Population	32.95 (1)	<0.0001	17.40 (1)	<0.0001	60.21 (1)	< 0.0001
Time period	165.96 (11)	<0.0001	93.09 (5)	<0.0001	92.82 (5)	< 0.0001
Clutch age	0.02 (1)	0.9010	0.28 (1)	0.5992	0.27 (1)	0.6041
Egg laying date	4.04 (1)	0.0445	0.79 (1)	0.3728	8.36 (1)	0.0038
**Female share**						
Population	8.67 (1)	0.0032	16.02 (1)	< 0.0001	3.89 (1)	0.0487
Time period	829.30 (11)	<0.0001	76.06 (5)	< 0.0001	264.82 (5)	< 0.0001
Clutch age	0.33 (1)	0.5654	1.31 (1)	0.2531	5.90 (1)	0.0151
Egg laying date	0.70 (1)	0.4025	3.80 (1)	0.05121	1.57 (1)	0.2104

Analysis for the full day (0–24 h), daytime (6–18 h) and night (18–6 h) data are shown separately.

Notes.

The full models included time period, clutch age, egg laying date as fixed terms and population random intercept term. The significance of each predictor was assessed by eliminating it from the full model and comparing the fit of the two models using likelihood ratio test.

### The effects of environment

Ambient temperature had a highly significant influence on both total incubation and female share (Fig. S1A, S1B, Table 2). However, the effect of temperature on both variables differed between time periods as indicated by the significant interaction between temperature and period (Table 2). In addition, the model estimating slopes for each population separately fitted the data better than the model fitting only a separate intercept for each population (Table 2, Fig. S1A, S1B) suggesting different population responses to ambient temperature.

The latter effect, however, is due to one population in each analysis (Ceuta in total incubation and Great Salt Lake in female share). By removing these populations from the respective analyses, the random slope term was no longer significant (total incubation: χ^2^ = 5.60, df  = 5, p = 0.3469; female share: χ^2^ = 4.14, df  = 5, p = 0.5296). The latter results suggest that plovers in all populations (except the two aforementioned ones) respond to ambient temperature in a consistent manner. Breeding site was a significant predictor of female share at night: island populations exhibit significantly less female share at night than mainland populations (β (SE)  = −0.41 (0.12), Table 2).

### The effects of life history

Clutch age had no influence on total incubation, nor on the female's share of daytime incubation (Table 3). However, with increasing clutch ages females tend to incubate more at night (β (SE)  = 0.02 (0.01), Table 3). Since % total incubation at night was unrelated to clutch age (Table 3), males appear to decrease their share of incubation with clutch age.

Clutches laid late in the season were incubated less at night than early clutches (β (SE)  = −0.05 (0.02), Table 3), although both the male and the female appear to decrease incubation time, since no significant association was found between laying date and % female share (Table 3). These life-history predictors of incubation behaviour remained significant when environmental variables were included in these models (results not shown).

### Genetic differentiation

To test whether genetic differentiation between Kentish and snowy plovers may influence incubation behaviour, we added species as a random factor to the models of both environmental and life-history variables. Nevertheless, including the species factor in the models did not improve fit in any of these models (likelihood ratio tests, all p>0.9, results not shown).

**Figure 3:**
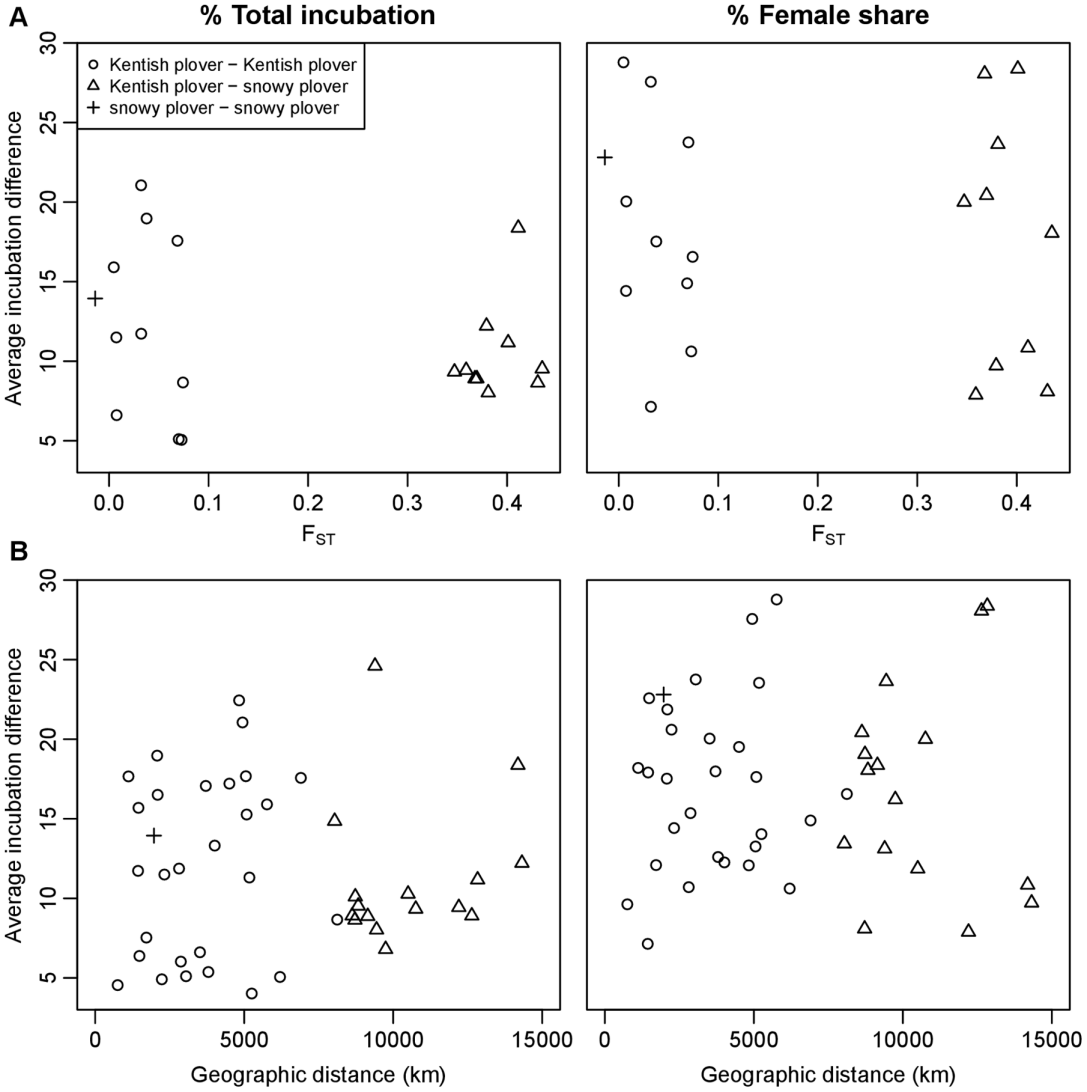
Pairwise differences in total incubation and female share of incubation between plover populations in relation to pairwise FST values estimated using 21 autosomal microsatellite markers (A), and pairwise geographic distances between populations (B).

Consistently, genetic differentiations between populations were unrelated to pairwise differences in both total and female share of incubation (Mantel-tests, % total incubation: z = 45.17, p = 0.284; % female share: z = 74.08, p = 0.769, Fig. 3A). Geographic distances between populations were also unrelated to pairwise differences in total incubation and female share (Mantel tests, % total incubation z = 3.2×10^6^, p = 0.903; % female share z = 4.6×10^6^, p = 0.945, Fig. 3B).

Furthermore, behavioural differences among Kentish plover populations remain unrelated to F_ST_ (Mantel tests, % total incubation z = 4.56, p = 0.535, % female share z = 6.87, p = 0.584) and to geographic distance (% total incubation z = 1.28×10^6^, p = 0.632, %female share z = 1.76×10^6^, p = 0.501).

Finally, to control for the significant ambient temperature effect between sites, we repeated the preceding analysis using residuals of the environmental model (full day models in Table 2). Nonenetheless, neither genetic nor geographic distance predicted temperature corrected residual % total incubation and % female share (all p>0.2, results not shown).

## Discussion

The striking diversity of parental care has long intrigued evolutionary biologists, and there is no single explanation for the evolution of parental cooperation that would apply to a wide range of taxa (2,9,10,16,22,39]. Identifying the ecological, life-history and genetic correlates of cooperation between two, usually unrelated parents, has a key importance in understanding sex roles and breeding system evolution [2], [13], [39]. Here we carried out a study to identify such factors that potentially influence biparental care in small plovers over an unusually large breeding range.

### Environmental and genetic effects on biparental care

Our study provided three key results. First, we showed that both total incubation and female share of incubation are significantly different between plover populations, and these differences are persistent throughout the day.

Second, we found a strong influence of ambient temperature on both total incubation and the female share. Consistent with previous studies [14], [43], the effect of temperature on total incubation was quadratic and depended on time of the day (Fig. S1A). Together, these results suggest that the parents need to balance keeping egg temperature within the optimal embryonic development against their own physiological requirements (e.g. feeding in the morning and late afternoon). Optimal embryonic development occurs in a narrow range of egg temperature, ranging from 36°C to 40.5°C in most bird species [54]. Temperatures below this optimum (hypothermia) are associated with slowed development, and prolonged exposure leads to embryo mortality or developmental disorders. Hyperthermia is even more problematic than hypothermia, since embryonic mortality rates increase sharply with egg temperatures above 40.5°C [54]. Keeping the eggs in the optimal thermal interval has direct fitness consequences, and incubation behaviour should be adjusted to optimize egg temperatures, therefore an increased parental investment is crucial in suboptimal ambient conditions. The harsher the environment, the more important parental care becomes, either to warm the eggs in cold weather, or to cool them in hot conditions. Kentish and snowy plovers nest in small scrapes on the ground, with usually little or no cover [14], [23]. This exposes the eggs more to solar radiation and eggs will overheat faster than those of species that nest in the shade or in protected sites such as tree holes and rock cavities.

The significant time period × temperature interaction suggests that parents respond differently to ambient temperature depending on the time of the day. Although the temperature range that the nesting plovers are exposed varies between populations, there is an overall distinctive pattern for each 2-hour time period that fits most populations (Fig. S1A, S1B). This striking result suggests phenotypic plasticity: plovers in most populations appeared to respond in a consistent manner to ambient temperature within time periods. Different incubation patterns over the course of the day and the significant population differences once temperature has been controlled, suggest that not only ambient temperature, but other environmental and genetic factors may also modulate incubation behaviour. For instance, parent birds may be locally adapted to meet their metabolic needs and cope with parasites, predators and other biotic and abiotic variables to adjust incubation behaviour to a standard diurnal activity.

Third, we found no effect of genetic differences (estimated by presumably neutral markers between populations) on total incubation or female share. These results were consistent with the non-significant effect of species and geographic isolation on incubation behaviour, and indicate that parental behaviour, at least among closely related plover species, is flexible and responds to local environment.

We propose three explanations for these results. (i) Within species gene flow may be high between geographically distinct populations, and strong mixing occurs between distant plover populations over large geographic distances [48], [49], [55]. Therefore genetic differences between populations may not be large enough to have a detectable effect on incubation behaviour. However, some plover populations are genetically distinct, for instance, breeding Kentish plover populations in the Farasan Island and Cape Verde are genetically differentiated from the mainland Kentish plover populations [49]. Although genetically distinct, these populations showed broadly similar responses to ambient temperature. Therefore, the low genetic separation between populations alone does not seem a plausible explanation. (ii) Behavioural differences may arise as a result of genetic differences in genes not studied here, e.g. variation in coding sequences, rather than differences in our presumably neutral genetic markers [20], [56]. Since coding and non-coding DNA sequences may be subject to different mutational and selective processes and since mutations in a single gene can have profound effects on phenotypes [40], [57], we cannot exclude the explanation that plover populations differ in genes related to parental care. To identify relevant genetic variants influencing parental care a detailed genome-based approach is needed. (iii) Although environmental contribution to phenotypic plasticity is spectacular [12], [40], [57], our statistical models of gene and behaviour associations did not test for possible environmental effects on gene expression (i.e. gene × environment interactions). Differences in phenotype might well be the result of transcriptional or post-transcriptional level modifications and/or of epigenetic modulations of gene expression driven by the social (or ecological) environment.

### Biparental care and harsh environment

Our results support the harsh environment hypothesis, because parental cooperation increases as ambient temperature leaves optimal egg development temperature ranges, when offspring survival appears to depend more on the care provisioned. When the eggs are exposed to overheating, the total incubation reaches almost 100% of time and incubation is shared approximately equally between males and females.

Our results suggest that as the environment moves away from the optimal embryonic development, (e.g. toward cold or hot temperatures), male contribution becomes essential to protect the eggs especially during the challenging parts of the day (e.g. during the day females become constrained by their ability to cope on their own with heat stress). This is concordant with theoretical models, which predict high level of cooperation when one parent cannot cope with the costs of rearing alone leading to social monogamy and long-term pair bonds [16], [39], [58]. With effective parental cooperation parents can defeat heat stress, which they would not be able to do alone and protect the offspring from hyperthermia at the same time [14], [23]. Therefore, high ambient temperatures may limit the opportunities for a sexual conflict over incubation [23]. Biparental care thus has obvious direct fitness consequences both in terms of survival and reproduction in an environment where harsh conditions occur even if on an irregular basis.

Plover populations exhibit variation in the extent of biparental care, and these behavioural differences are predicted by the local environment, but not by genetic differences in non-coding genetic markers. We propose that phenotypic plasticity exhibited by adults is a likely explanation for the different behaviours exhibited by plover populations. Phenotypic plasticity, in turn, may be a key facilitator of the unusually wide ecological and geographic range of breeding plovers, and of associated adaptations to the local environments. The latter results are consistent with recent works that show large gene exchange between geographically different plover populations [48], [49], [55], and thus emphasize the significance of phenotypic responses to local environment.

In conclusion, our study provides evidence that environment plays a crucial role in the evolution of biparental care on a large geographical scale by showing that harsh environmental boosts cooperation among genetically unrelated parents. Although ambient temperature influences biparental care at least during incubation, further studies are needed to test the influences of additional social and asocial factors on parental behaviour, and extend the scope to post-incubation care including the care of hatchlings, fledglings and post-fledged young. Taken together, these studies will reveal how males and females balance the cost and benefits of care leading to conflicting interests and/or parental cooperation.

## Supporting Information

Figure S1 **Predicted (a) total incubation and (b) female share of incubation in relation to ambient temperature over 12 time periods of the day in different plover populations (see** **Table 3** **).** Number of nests observed in each time period are given in the legend.
(PDF)

Table S1 **Mean (and standard deviation) in % total incubation, % female share and ambient temperature, number of nests, number of two-hour records and the years of data collection are shown for each population.** In total, the study includes 1628 records from 285 plover nests.
(DOC)
