# Supplementary figures and images for: Local Environment but Not Genetic Differentiation Influences Biparental Care in Ten Plover Populations
Source: PLoS One. 2013 Apr 17;8(4):e60998. doi: 10.1371/journal.pone.0060998 (PMC3629256; doi:10.1371/journal.pone.0060998)

A

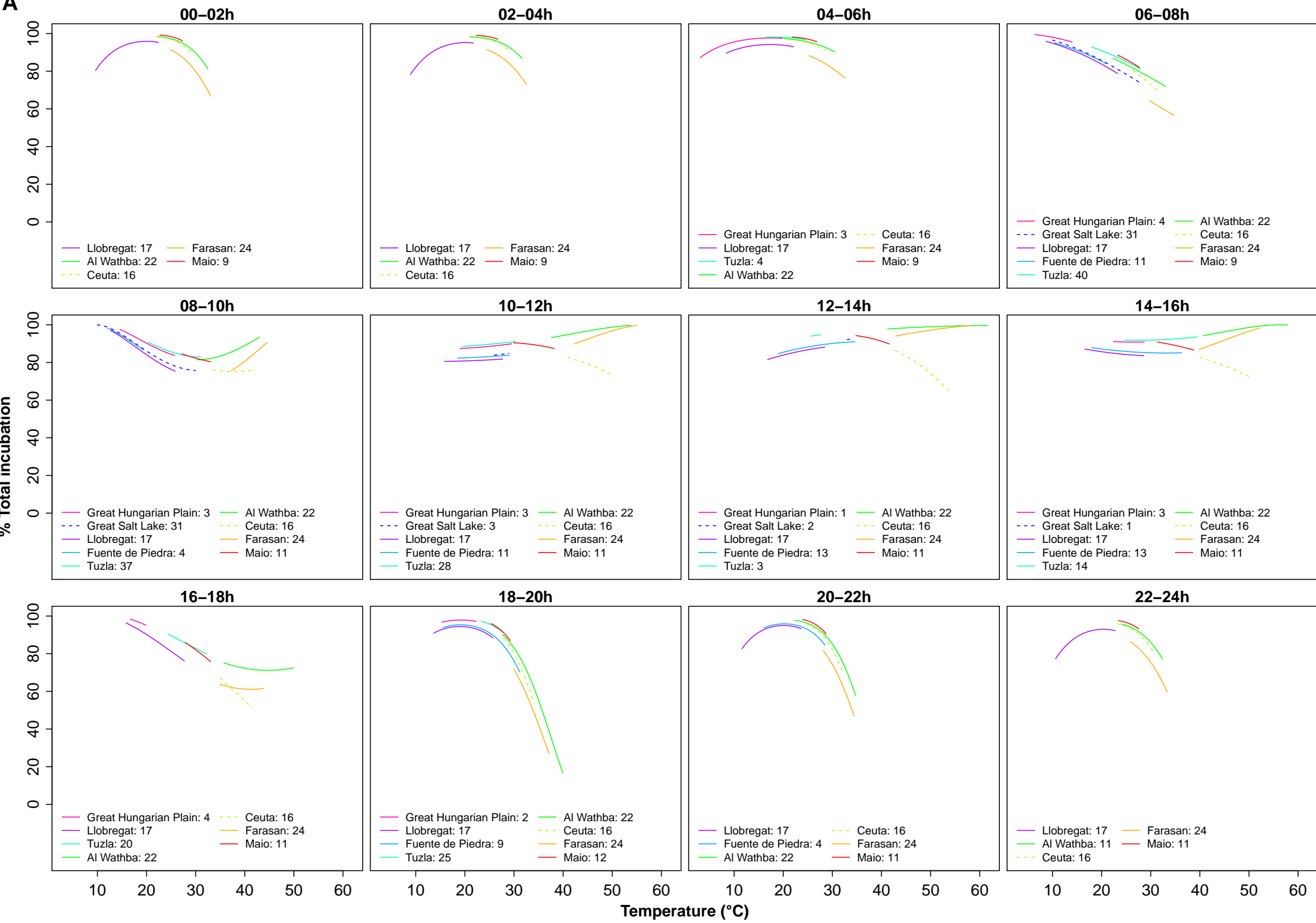

**B**

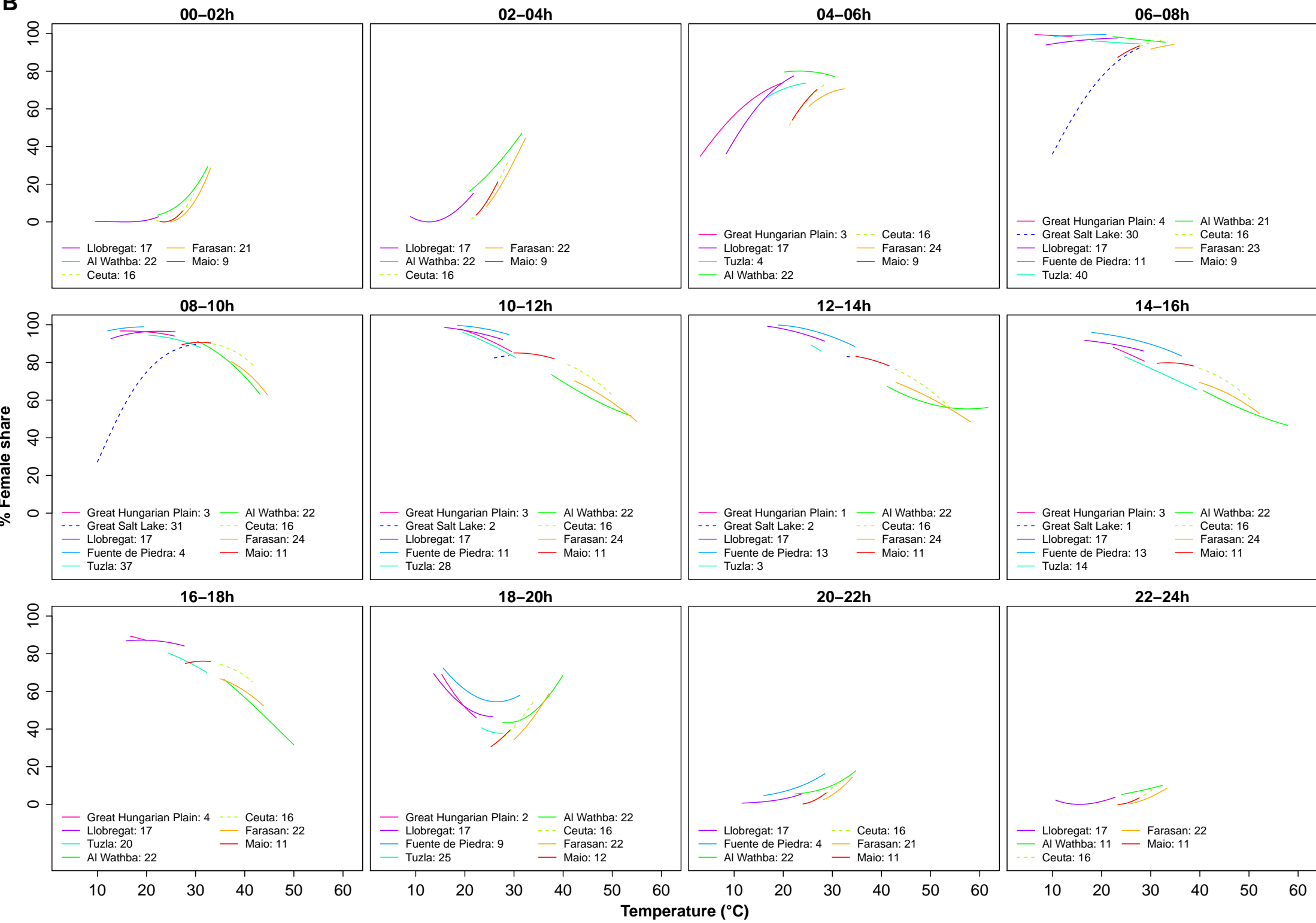

Supplement: Figure S1 — Predicted (a) total incubation and (b) female share of incubation in relation to ambient temperature over 12 time periods of the day in different plover populations (see Table 3 ). Number of nests observed in each time period are given in the legend. (PDF) [file pone.0060998.s001.pdf]
