# Supplementary material for: Local Environment but Not Genetic Differentiation Influences Biparental Care in Ten Plover Populations
Source: PLoS One. 2013 Apr 17;8(4):e60998. doi: 10.1371/journal.pone.0060998 (PMC3629256; doi:10.1371/journal.pone.0060998)
Supplement: Table S1 — Mean (and standard deviation) in % total incubation, % female share and ambient temperature, number of nests, number of two-hour records and the years of data collection are shown for each population. In total, the study includes 1628 records from 285 plover nests. (DOC) [file pone.0060998.s002.doc]

**Table S1.**

| Population | Species | Total incubation (%) | Female share (%) | Ambient temperature (^◦^C) | No. nests | No. records | Years |
| --- | --- | --- | --- | --- | --- | --- | --- |
| 1 | Kentish plover | 99.23 ± 1.05 | 59.06 ± 21.77 | 10.2 | 7 | 49 | 1955 |
| 2 | Kentish plover | 86.13 ± 9.84 | 60.35 ± 16.39 | 18.25 ± 5.19 | 47 | 93 | 1988-1992 |
| 3 | Kentish plover | 85.81 ± 3.97 | 47.02 ± 34.09 | 18.87 ± 2.78 | 17 | 204 | 2005-2008 |
| 4 | Kentish plover | 83.5 ± 6.61 | 69.74 ± 10.71 | 22.82 ± 5.12 | 14 | 70 | 1997-1998 |
| 5 | Kentish plover | 88.34 ± 5.84 | 47.76 ± 31.76 | 25.63 ± 2.88 | 83 | 239 | 1997-1999 |
| 6 | Kentish plover | 85.03 ± 15.42 | 41.64 ± 21.32 | 35.22 ± 9.17 | 22 | 253 | 2005-2006 |
| 7 | Kentish plover | 75.44 ± 16.71 | 35.65 ± 19.01 | 37.23 ± 8.97 | 24 | 288 | 2008-2009 |
| 8 | Kentish plover | 88.05 ± 7.75 | 42.77 ± 28.36 | 29.31 ± 5.08 | 14 | 159 | 2009 |
| 9 | Snowy Plover | 82.87 ± 11.95 | 67.31 ± 18.4 | 27.25 ± 6.46 | 41 | 81 | 2008-2009 |
| 10 | Snowy Plover | 79.49 ± 9.15 | 38.39 ± 22.77 | 34.07 ± 8.46 | 16 | 192 | 2006 |
